# The Role of a First Aid Training Program for Young Children: A Systematic Review

**DOI:** 10.3390/children10030431

**Published:** 2023-02-23

**Authors:** Eleana Tse, Katerina Plakitsi, Spyridon Voulgaris, George A. Alexiou

**Affiliations:** 1 Department of Neurosurgery, School of Medicine, University of Ioannina, 45500 Ioannina, Greece; 2 Department of Early Childhood Education, School of Education, University of Ioannina, 45500 Ioannina, Greece

**Keywords:** first aid, training, children, systematic review

## Abstract

Background: Many first aid programs have been conducted in schools, and researchers have identified that interventions improved students’ knowledge, skills, and attitude. This study examines the content, practices, and assessment of first aid interventions at primary schools and evaluates their effectiveness. Methods: A systematic review was undertaken. We searched MEDLINE and Cochrane library databases from January 1990 to December 2021 using the search terms: ‘’first aid’’ AND ‘’primary school children’’. School-based first aid training targeting 6 to 10 years old studies in English were eligible for inclusion. Results: We included 11 studies that were approached by experimental (n = 6) and by observational studies (n = 5). Researchers conducted interventions in Europe (n = 9) and America (n = 2). An essential part of the teaching was hands-on practice. Most studies included in their program cardiopulmonary resuscitation (n = 8) and basic life support (n = 7). The main findings showed that trained children have significantly better knowledge of and skills in first aid than those before or without training. Children under 11 years old were not strong enough to achieve the proper depth of chest compressions. Depth of chest compressions correlates with children’s age, weight, height (n = 2), and body mass index (n = 3). Conclusions The effectiveness of resuscitative or non-resuscitative first-aid training for primary school children improved students’ knowledge and skills. Subsequent research could investigate children’s reactions in actual first aid conditions.

## 1. Introduction

Every human life is valuable and must be safeguarded. Countries go to great lengths to protect their citizens from natural disasters and other threats, but millions of people die each year. One of the leading causes of death is cardiac arrest [1,2]. Daily, a large number of people die as a result of cardiac arrest [2]. This number would be lower if more people were trained in first aid and provided immediate assistance to people facing cardiac arrest, as the brain can survive without oxygen for 3–5 min. As a result, learning first aid is critical because it can help people to respond quickly to emergency situations and save lives.

First aid training is necessary for every person of every age since both severe and trivial accidents happen in life. People who have been trained in first aid can save lives [1]. The Red Cross gives training programs offering resuscitative and non-resuscitative lessons. These programs aim to increase the number of trained bystanders. It is estimated that at least 15% of the population have to be trained in order to achieve a statistically significant increase in resuscitation results, and this number cannot be achieved through voluntary courses [1]. The school is an environment where a significant number of people can be trained at a low cost [1,2,3,4,5,6,7,8,9,10,11,12,13].

In addition, researchers are exploring expanding first aid training programs to, targeting first aid education for young children [2,3,4,5,6,7,8,9,10,11,12,13]. Several studies conducted in developed countries illustrate that children can acquire first aid skills through education, increasing their knowledge and practice in life-saving situations [2]. The scope is to increase the number of bystanders by cultivating a positive attitude toward helping people in need from an early age [1]. Researchers identified that interventions improved students’ knowledge, skills, and perspective in performing first aid [3,4,5,6,7,8,9,10,11,12,13]. The World Health Organization approved training in cardiopulmonary resuscitation for school-age children in 2015 [2], and endorsed the European Resuscitation Council (ERC) campaign “Kids Save Lives”, which emphasized the importance of including basic life support (BLS) education in school curricula [2]. By including BLS education in schools, this information can reach a large portion of the population, potentially increasing bystander CPR rates and survival rates significantly.

Children do not learn in the same manner that adults do; therefore, training materials should be specifically designed and adjusted to make them quite accessible to young people in order to foster meaningful learning and enhance not only students’ understanding but also knowledge retention over time. The present study aims to examine published articles that have studied the training of primary school children in first aid. The research questions that this systematic study attempts to answer are:

1. What is the content of the first aid interventions at primary school?
2. What are the standard practices and assessments of first aid interventions?
3. What is the current evidence for the effectiveness of interventions?

## 2. Materials and Methods

The current systematic review followed the PRISMA (Preferred Reporting Items for Systematic Reviews and Meta-Analyses) guidelines [14]. All authors agreed on the methodology used, which included screening the title and abstract, full text reading, inclusion/exclusion, and data extraction criteria. A review protocol was submitted to PROSPERO, a database of systematic review protocols (ID CRD42023393720).

### 2.1. Search Strategy

Two reviewers (ET, GA) conducted an independent database search for internal validity. The following combination of search strings was used in both database (MEDLINE and Cochrane library) searches: “first aid” AND “primary school children” (Appendix A). The last literature search was conducted on 3 January 2022. Next, we searched the references of identified articles for additional citations. The title and abstracts identified in the data search were screened to assess adherence to the search criteria. We filtered the full text to determine inclusion when the abstract did not provide sufficient information.

### 2.2. Search Eligibility

Studies were eligible for inclusion if there was school-based first aid training targeting primary school children, 6 to 10 years old. The inclusion criteria were predefined and finalized before the search. We used the following inclusion and exclusion criteria for the selection of articles: (1) Population: Included only primary school children, 6 to 10 years old. Children with disabilities were excluded, (2) Intervention: We included studies on first aid education in the following topics: calling the emergency number, choking, automated external defibrillation (AED), bleeding, cardiopulmonary resuscitation (CPR), assessment of consciousness, and stings and bites. Asthma, mental health, epilepsy, burn prevention and teeth were excluded, (3) Type of article: We excluded surveys, letters, and systematic reviews, (4) Language: We only included studies in English, and (5) Assessment: We included studies in which the training and evaluation was for children, targeted in primary schools, aged 6 to 10 years.

### 2.3. Data Extraction

The extracted data related to study design, population (number of participants and age range), characteristics of educational program (content, facilitator, duration, assessment) and outcomes. Two reviewers (ET, GA) independently assessed each article for internal validity. Differences were resolved involving the second author (KP).

### 2.4. Study Quality Assessment

We used the NIH Study Quality Assessment for observational studies and controlled intervention studies [15]. We responded to each question with a ‘yes’, ‘no’, or ‘other’. We made a decision based on the overall assessment.

## 3. Results

**Figure 1:**
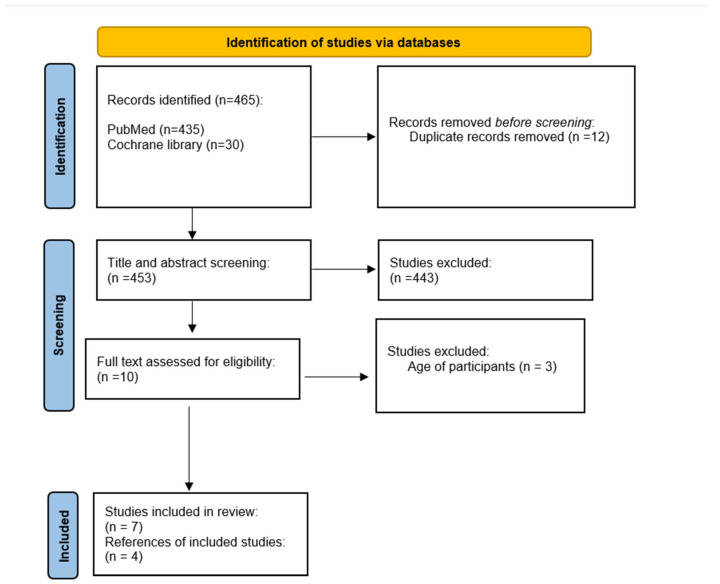
Prisma flow diagram.

Based on the search strategy described, 465 articles were initially identified (Figure 1). We excluded 458 articles and included seven articles. Of the excluded articles, we excluded 12 based on duplicates and 443 based on the title and abstract review (for example, studies that examined teachers’ knowledge) and three based on the full paper review (the reason for exclusion was the target age of participants). Four articles were also added from the references of the included articles. Thus, the present review included 11 articles. From the included articles (Table 1), two examined students aged 6 to 7 years [3,5], two examined students aged 10 to 11 years old [8,11], three looked at students aged 10 to 12 years old [10,12,13], one examined students aged 8 to 11 years [4], one examined students aged 7 to 14 years [6], one examined students aged 9 to 18 years [7] and one examined students aged 9 to 14 years [9]. Nine included articles were conducted in Europe [3,4,5,6,7,8,10,11,12] and two in America [9,13]. In the study design, six were an experimental study [3,4,8,10,11,13] and five an observational study [5,6,7,9,12].

The content of the first aid programs was varied (Table 2). Eight studies included cardiopulmonary resuscitation in their program [5,7,8,9,10,11,12,13], seven studies included basic life support [4,6,7,8,9,10,12], five studies had emergency calls [3,5,6,7,8], four studies included bleeding [3,4,5,6], three studies included automated external defibrillation [5,6,7], three studies included recovery position [3,5,7], two studies included burns [5,8], and one study included wound treatment [3]. One study included electrocution [8]. Resuscitative programs were the focus of five studies [9,10,11,12,13], non-resuscitative programs of one study [3], and both of five studies [4,5,6,7,8].

The duration of the programs varied from 1 to 5 days. Four interventions lasted one day [9,10,11,12], three interventions lasted three days [4,6,13], two interventions lasted five days [3,5], and two studies did not specify the duration [7,8]. The duration per session varied from 20–360 min. Five studies lasted 120 min/session [5,10,11,12,13], two studies lasted 45 min/session [3,6], one study lasted 360 min/session [7], one study lasted 20 min/session [9] and two studies did not specify the duration per session [4,8].

Most first aid programs (n = 5) were taught by first aid professionals [3,4,6,9,13]. Three studies used teachers to teach first aid [7,10,12]. Two studies used professionals and teachers [5,8], and one did not specify the instructor [11].

An essential part of the teaching was hands-on practice, using manikins (n = 7), video (n = 3), slide projector (n = 1), tour of the emergency department (n = 1), and glove puppet/simulators (n = 1).

The evaluation tools which researchers used to assess the children’s knowledge, skills, and attitudes were questionnaires (n = 7), test scenarios (n = 3,) and practice with manikins (n = 4). Most studies (n = 7) assessed children’s knowledge, skills, and attitudes pre- and post-intervention. Seven studies assessed children’s knowledge and skills pre-and post-intervention [3,5,6,8,10,12,13], four studies assessed post-intervention [4,7,9,11] and four studies had follow-up assessment [3,4,6,10].

The main findings of the included studies (Table 3) were that trained children have significantly better knowledge and skills than those before (n = 4) or without training (n = 2). In addition, children under 11 years old were not strong enough to achieve the proper depth of chest compressions (n = 3), and the depth of chest compressions correlates with children’s age, weight, height (n = 2), and body mass index (n = 3). One study showed that children with practical training have significantly better knowledge than those without practical training [4]. One study identified that children achieve greater depth of chest compressions when using a ratio of 15:2 rather than 30:2 [11].

## 4. Discussion

The present systematic review identified 11 studies implementing a first aid program in primary schools. The results of the included articles showed that trained children have significantly better knowledge than children without training [3,4,5,6,8,10,12]. Children under the age of 11 cannot properly apply chest compressions or the tidal volume for mouth-to-mouth resuscitation [6,7,8,13]. Chest compression depth was found to be significantly related to children’s age, weight, height, and body mass index [6,9].

The studies varied in their programs. Most studies trained children in cardiopulmonary resuscitation, basic life support, and emergency calls. This finding partially agree with Reveruzzi et al. [16], who found that most studies were mainly limited to teaching basic life support, CPR, and AED skills. Children are a vulnerable group. Because they are not mentally and physically mature, they are more likely to be injured, particularly at school [17]. They are more vulnerable to accidents and injuries because of their participation in games and other social activities. Teaching young students in school and kindergarten can be an effective way to develop first aid capabilities for children of a younger age in order to prevent serious injuries from school accidents. School and kindergarten accidents can be severe, with research showing that the most injured part of the human body at such young ages is the head. Head injuries are a common type of injury in our daily lives, particularly among children [17]. Head injuries are a common reason for hospitalization and can result in disability or death at any age. Minor traumatic brain injury is the most common. Our impression is that no research has dealt with head injuries, even though most children’s injuries in kindergarten and primary schools are in the head [17]. These studies have focused on resuscitative programs. This finding conforms to the review of He et al. [18] and Tse et al. [19], who mention that most first aid programs focus on CPR.

Findings confirmed longer-duration programs with both practical and didactic components. Several studies with at least 3 h of lesson time reported significant improvements in knowledge and information. This finding is consistent with Reveruzzi et al. [16].

Our systematic review identified that children under 10 years old were not strong enough to achieve the proper depth of chest compressions. This finding is in keeping with other studies which conclude that children older than ten years old could perform cardiopulmonary resuscitation properly [16]. Chest compression depth was found to be significantly related to children’s age, weight, height, and BMI. The same factors influenced ventilation [6,7,9].

The results of studies using a variety of facilitators to teach first aid to young children showed that both first aid instructors and teachers had beneficial effects on their students’ first aid knowledge, skills, and attitude [20]. It is encouraging that teachers may deliver first aid instruction if they have received the necessary first aid training. Since it is a cost-effective way to increase the number of bystanders, using teachers as facilitators could result in lower costs [10,12]. The systematic review showed that most researchers selected first aid professionals as training facilitators in practice and assessment of first aid interventions. It is interesting to compare the outcomes of teachers versus first aid professionals.

Studies used teaching hands-on training, as reported by Reveruzzi et al. [16]. These studies showed that practical components in the education process delivered better results than purely theoretical training [19]. Moreover, most studies assessed children’s knowledge, skills, and attitudes pre- and post-intervention, and few studies followed up at 12 months post-intervention, following the review of He et al. [18]. Finally, an exciting outcome relating to the effectiveness of the reviewed programs is that trained children showed significantly better knowledge and skills than the same children before training or other untrained children.

Despite the fact that the research findings show that teaching first aid should begin at a young age [20,21,22], several research questions remain unanswered, including the length of time that first aid training knowledge is retained in children’s memories, which instructor is more effective (first aid experts or teachers), when the program should be repeated, and whether the intervention program is actually effective in improving students’ knowledge, skills, and attitudes toward first aid.

Another important factor to consider when researching teaching first aid in primary school is students’ reactions in real-life first aid situations following participation in educational programs. As a result, it is necessary to assess how the children would react in real-life scenarios, as well as which aspects of training should be prioritized and intensified in order to improve students’ effective reactions in real-life situations. As a result, research should focus on real-world scenarios to provide data that can be used to improve the effectiveness of the first aid training. However, knowledge of first aid also influences first aid attitudes. The willingness to provide initial care may rise with first aid training. Numerous studies support the idea that instructing children and teenagers in first aid and cardiopulmonary resuscitation (CPR) could boost people’s willingness to provide effective assistance in emergency situations [23].

A carefully thought-out educational first aid program should be implemented. Scenarios and practice must be based on actual events in order that the learner understands them. Instructors must use a variety of teaching strategies to help students learn new information. Reder et al. [24] compared two new instructional methods, interactive-computer training, and interactive-computer training plus instructor-led (hands-on) practice, to traditional classroom instruction, which included video, teacher demonstration, and instructor-led (hands-on) practice, as well as a control group. All groups had an average knowledge score of more than 75%, and students in the computer-based training and tutored practice groups outperformed those in the traditional classroom instruction group. Students who received hands-on practice performed CPR better than those who only received computer training. Role-playing, hands-on training, the use of technology, and the execution of realistic scenarios are the foundations of educational first aid programs [3,4,5,6,7,8,9,10,11,12,13].

After first aid training, knowledge evaluation questionnaires have been the only means of determining the proficiency of first aid skills. Questionnaires have been developed by researchers. Questions that were either multiple-choice, open-ended, or scenario-based were used to measure the depth of knowledge. However, in most cases, this type of assessment is combined with the assessment of practical skills. Resuscitation training manikins are widely used to assess the efficiency of BLS skills (CPR/AED). This finding agrees with Minna et al. [25], who found that the most commonly first aid skills after first aid training were practical skills, specifically the ability to perform cardiopulmonary resuscitation (CPR) and use an automated external defibrillator. This assessment was based on several standardized measurements and was frequently performed with the assistance of a resuscitation manikin and an observer. In other emergency situations, questionnaires used to assess first aid knowledge were not standardized.

School is a place where many people can be educated at a low cost. Students and teachers can play a role as “multipliers” for the first aid training program, increasing the number of people who are trained in first aid [26,27,28]. To significantly reduce deaths from cardiac arrest, it is estimated that at least 15% of the population will need to be trained in CPR [1]. This percentage cannot be achieved solely through the voluntary offering of courses by trainees or trainers. As a result, the school is an ideal location for first aid training [29].

It is crucial to regularly teach first aid in schools [30,31]. School is a good location to teach first aid since it can reach many people and educate them efficiently. Increasing the number of onlookers will decrease the number of fatalities [32]. Once more, teaching first aid to kids at a young age could foster the social responsibility that society needs in order to advance [19,30,31,32,33,34,35]. Educating children in school helps them not only know how to handle an accident, but also how to handle crises, as accidents and unpleasant events occur frequently in the family and school environment [35]. To sum up, we firmly feel that teaching young children both resuscitative and non-resuscitative first aid is essential [34,36,37,38,39,40].

## 5. Limitations

There are two major limitations to the current study. One of the limitations of the review is language. It is possible that other studies have been conducted on this topic and reported in a language other than English. Another limitation is the database. We used only two databases. It is potential that studies on this subject have been carried out and published in a different database.

## 6. Conclusions

This is the first systematic review of first aid training in ages 6–10 years. This article explores first aid programs at primary school and provides information about content, practices, assessment, and the effectiveness of interventions. Training young students in school and kindergarten can be an effective way to develop first-aid skills and prevent serious injuries from accidents. Most studies taught children cardiopulmonary resuscitation and emergency calls, using hands on training. Nevertheless, children under the age of 10 were unable to achieve the required depth of chest compressions. Children can learn cardiopulmonary resuscitation but cannot perform chest compressions. There was a significant relationship between chest compression depth and children’s age, weight, height, and body mass index. The same factors affected ventilation. Moreover, one important finding is that teachers who have received the necessary first aid training may provide first aid instruction. Using teachers as facilitators may help to reduce costs. The review indicates that resuscitative and non-resuscitative first-aid training for children improves students’ knowledge and skills in non-real first-aid conditions. Subsequent research can investigate children’s reactions in actual first aid conditions. To conclude, the present study discovered that first aid programs help students improve their knowledge and skills. Nevertheless, numerous aspects have not been investigated, making scientific and reliable results impossible, as described previously.

## Figures and Tables

**Table 1:** Characteristics of included studies.

Author, Year	Study Design	Number of Participants	Age	Continent	Quality Assessment
Bollig et al., 2009 [3]	Experimental study	228	6–7	Europe	Good
Lubrano et al., 2005 [4]	Experimental study	469	8–11	Europe	Good
Uray et al., 2003 [5]	Observational study	47	6–7	Europe	Good
Banfai et al., 2017 [6]	Observational study	582	7–14	Europe	Good
Fleischhackl et al., 2009 [7]	Observational study	151	9–18	Europe	Good
Frederick et al., 2000 [8]	Experimental study	1200	10–11	Europe	Good
Jones et al., 2007 [9]	Observational study	157	9–14	America	Good
Connolly et al., 2007 [10]	Experimental study	79	10–12	Europe	Good
Hill et al., 2009 [11]	Experimental study	85	10–11	Europe	Good
Toner et al., 2007 [12]	Observational study	190	10–12	Europe	Good
Berthelot et al., 2013 [13]	Experimental study	80	10–12	America	Good

**Table 2:** Educational program.

Author, Year	Instructor	Lessons	Duration per Session (min)	Content of Program	Evaluation Tool	Educational Material
Bollig et al., 2009 [3]	First Aid Professional	5	45	B, F, C, B, WT, BL, U, OA, RP, BIES, EC	Test scenario	Glove puppet
Lubrano et al., 2005 [4]	First Aid Professional	3	45	BT, NB, PBLS	Multiple- choice & semi- structured test	slide projector & two pediatric simulators
Uray et al., 2003 [5]	First Aid Professional & teachers	5	120	EC, CPR, AED, RP, BL, BU	Questionnaire s & videotapes of the training	Game & manikins
Banfai et al., 2017 [6]	First Aid Professional	3	45	BLS, AED, U, BL, EC	questionnaire & observation	Scenario
Fleischhackl et al., 2009 [7]	Teachers	Not Provided	360	EC, CFVS, RP, CPR AED	Practice in Manikin	Manikins
Frederick et al., 2000 [8]	Teachers & first aid professionals	Not Provided	Not Provided	BLS, CPR BU, CU, E, FAR	Quiz & scenario	Video, tour of an accident & emergency department
Jones et al., 2007 [9]	First Aid Professional	1	20	BLS, CPR	Practice in Manikin	Manikin
Connolly et al., 2007 [10]	Teachers	1	<120	BLS, CPR	multiple choice questionnaire	Video & manikin
Hill et al., 2009 [11]	Not provided	1	120	CPR	Practice in manikin	Manikin
Toner et al., 2007 [12]	Teachers	1	120	BLS, CPR	multiple choice questionnaire	Video & manikin
Berthelot et al., 2013 [13]	First Aid Professional	3	120	CPR	questionnaire & Practice in manikin	Manikin

Abbreviations: AED: automated external defibrillation, B: breathing BLS: essential life support BMI: body mass index BU: burns C: consciousness CCD: chest compressions of depth CFVS: check for vital signs CO: collapse CPR: cardiopulmonary resuscitation CU: cuts D: defibrillation E: electrocution EC: emergency call ECI: emergency call information ECT: emergency call telephone FAR: first aid responses OA: open airway PBLS: pediatric essential life support RP: recovery position T: trauma.

**Table 3:** Findings of the study.

First Author, Year	Effect	Main Findings
Bollig, 2009 [3]	C, B, OA, RP, ECR, ECI (*p* < 0.001)	Trained children have significantly better knowledge than children without training.
Lubrano, 2005 [4]	PBLS (*p* < 0.001)	Trained children with practical training to have a considerably better understanding than trained children without suitable training.
Uray, 2003 [5]	CO, CPR, T, RP, BU, D (rose 20–25%)	Trained children have significantly better knowledge than before training.
Banfai, 2017 [6]	BLS, AED, U, BL, EC (*p* < 0.01)	Trained children have significantly better knowledge and skills than before training. There was a significant correlation between chest compression depth and children’s age, weight, height, and body mass index. Ventilation depended on the same factors.
Fleischhackl, 2009 [7]	EC (95% success rate), CFVS (85% success rate), RP (70% success rate), CPR (86% success rate), AED (93% success rate)	Trained children can successfully and effectively learn BLS skills. Age did not influence the depth of chest compressions or tidal volume of mouth-to-mouth resuscitation. The student’s BMI mostly controlled the depth of chest compressions or tidal volume during mouth-to-mouth ventilation.
Frederick, 2000 [8]	BLS, CPR, BU, CU, E, FAR (*p* < 0.01)	Trained children have a piece of significantly better knowledge, skills, attitudes, and behavior than children without training.
Jones, 2007 [9]	CCD (0% success rate)	Children (<11 years) were not strong enough to compress the chest to an adequate depth in simulated cardiopulmonary resuscitation. The compression depth is significantly associated with the pupils’ age, weight, and height.
Connolly, 2007 [10]	BLS, CPR (*p* < 0.001)	Trained children have significantly better knowledge than before training.
Hill, 2009 [11]	15:2 vs. 30:2 (*p* < 0.001)	Children achieve greater depth of chest compressions when using a ratio of 15:2 rather than 30:2
Toner, 2007 [12]	BLS, CPR (*p* < 0.001)	Trained children have substantially better ability than before training.
Berthelot, 2013 [13]	CCD (5% success rate)	Children (10–12 years) were not strong enough to compress the chest to an adequate depth in simulated cardiopulmonary resuscitation.

Abbreviations: AED: automated external defibrillation, B: breathing BLS: essential life support BMI: body mass index BU: burns C: consciousness CCD: chest compressions of depth CFVS: check for vital signs CO: collapse CPR: cardiopulmonary resuscitation CU: cuts D: defibrillation E: electrocution EC: emergency call ECI: emergency call information ECT: emergency call telephone FAR: first aid responses OA: open airway PBLS: pediatric essential life support RP: recovery position T: trauma.

## Data Availability

Upon request from the authors.

## Appendix A

Literature search performed on 3 January 2022, by two authors (ET, AG) using online scientific databases.

Literatures identified from publications between 1990 up to 2021 in various databases PubMed (N = 435), Cochrane library (N = 30) resulted in a total of 465 hits. After appropriate screening 11 articles were selected for final review and analysis. Overall screening process and results can be found in Figure 1. (PRISMA flowsheet).

**Table A1:** PubMed search 3 January 2022.

No.	Search Terms/Limits	Result
1	(First aid).m_titl.	37,000
2	(Primary school children).m_titl.	139,309
3	1 and 2	532
4	limit 3 to english language	506
5	limit 4 to yr = “1990–2021”	435

**Table A2:** Cochrane library search 3 January 2022.

No.	Search Terms/Limits	Result
1	(First aid).m_titl.	513
2	(Primary school children).m_titl.	108
3	1 and 2	31
4	limit 3 to english language	31
5	limit 4 to yr = “1990–2021”	30
